# Trust in genomic data sharing among members of the general public in the UK, USA, Canada and Australia

**DOI:** 10.1007/s00439-019-02062-0

**Published:** 2019-09-17

**Authors:** Richard Milne, Katherine I. Morley, Heidi Howard, Emilia Niemiec, Dianne Nicol, Christine Critchley, Barbara Prainsack, Danya Vears, James Smith, Claire Steed, Paul Bevan, Jerome Atutornu, Lauren Farley, Peter Goodhand, Adrian Thorogood, Erika Kleiderman, Anna Middleton, on behalf of the Participant Values Work Stream of the Global Alliance for Genomics and Health

**Affiliations:** 1 Society and Ethics Research, Connecting Science, Wellcome Genome Campus, Cambridge, UK; 2 Institute of Public Health, University of Cambridge, Cambridge, UK; 3 RAND Europe, Cambridge, UK; 4 National Addiction Centre, King’s College London Institute of Psychiatry, Psychology and Neuroscience, London, UK; 5 Centre for Epidemiology and Biostatistics, Melbourne School of Global and Population Health, The University of Melbourne, Melbourne, Australia; 6 Centre for Research Ethics and Bioethics, Uppsala University, Uppsala, Sweden; 7 Centre for Law and Genetics, University of Tasmania, Hobart, Australia; 8 Department of Statistics and Epidemiology, Swinburne University of Technology, Melbourne, Australia; 9 Department of Political Science, University of Vienna, Vienna, Austria; 10 Department of Global Health and Social Medicine, King’s College, London, UK; 11 Melbourne Law School, University of Melbourne, Parkville, VIC Australia; 12 Murdoch Children’s Research Institute, Parkville, VIC Australia; 13 Department of Public Health and Primary Care, Centre for Biomedical Ethics and Law, KU Leuven, Leuven, Belgium; 14 Leuven Institute for Human Genomics and Society (LIGAS), KU Leuven, Leuven, Belgium; 15 Web Team, Wellcome Sanger Institute, Wellcome Genome Campus, Cambridge, UK; 16 School of Health Sciences, University of Suffolk, Ipswich, UK; 17 Ontario Institute for Cancer Research, MaRS Centre, Toronto, ON Canada; 18 Centre of Genomics and Policy, McGill University, Montreal, QC Canada; 19 Faculty of Education, University of Cambridge, Cambridge, UK

**Keywords:** Data sharing, Public, Trust, Genome, Donation, Survey

## Abstract

Trust may be important in shaping public attitudes to genetics and intentions to participate in genomics research and big data initiatives. As such, we examined trust in data sharing among the general public. A cross-sectional online survey collected responses from representative publics in the USA, Canada, UK and Australia (*n* = 8967). Participants were most likely to trust their medical doctor and less likely to trust other entities named. Company researchers were least likely to be trusted. Low, Variable and High Trust classes were defined using latent class analysis. Members of the *High Trust* class were more likely to be under 50 years, male, with children, hold religious beliefs, have personal experience of genetics and be from the USA. They were most likely to be willing to donate their genomic and health data for clinical and research uses. The *Low Trust* class were less reassured than other respondents by laws preventing exploitation of donated information. Variation in trust, its relation to areas of concern about the use of genomic data and potential of legislation are considered. These findings have relevance for efforts to expand genomic medicine and data sharing beyond those with personal experience of genetics or research participants.

**Electronic supplementary material:**

The online version of this article (10.1007/s00439-019-02062-0) contains supplementary material, which is available to authorized users.

## Introduction

The future of genomic medicine and research relies upon the sharing of health and genomic data to facilitate large-scale analyses and support clinical interpretation of genetic variants (ACMG Board of Directors 2017; Raza and Hall 2017). There is scientific and policy support for data sharing, and efforts are ongoing to develop technical, ethical and legal solutions to connect genomic databases and make them more accessible for clinical and research purposes (Kaye et al. 2009; Siu et al. 2016; Birney et al. 2017; Borry et al. 2018). In addition, sharing genomic data relies on the support and involvement of members of the public from whom data are collected—whether as patients or research volunteers (Nuffield Council on Bioethics 2015; Kaye et al. 2018). In this context, the notion of trust is particularly important (Trinidad et al. 2010; Eckstein et al. 2018; Lawler et al. 2018). Trust relates to the ability of researchers, institutions and governance arrangements to realise benefits and manage or mitigate risks associated with data sharing, including to privacy and confidentiality (Shabani et al. 2014; Nuffield Council on Bioethics 2015). It can be conceptualised as a heuristic that is activated when people are faced with risks and need to make decisions or a ‘leap of faith’ with little or no available information (Luhmann 2000; Mollering 2001; Lipworth et al. 2009).

Trust is consistently identified as an important factor both shaping attitudes to genomics and the intentions of members of the public to participate in genomics research and big data initiatives (Lipworth et al. 2009; Critchley et al. 2015; Nicol et al. 2016; Lawler et al. 2018). Conversely, the failure of the UK’s clinical data sharing initiative, care.data, has been partly attributed to a failure to obtain public trust (van Staa et al. 2016). Lack of trust in the initiative seems to be related to concerns about transparency in the use of data, respect for confidentiality and privacy and commercialisation (Sterckx et al. 2016). Where trust is absent, the social licence and mandate of researchers and clinicians to obtain and distribute data may be lost (Carter et al. 2015).

The collection and subsequent sharing of genomic data involves a range of actors, including individual doctors, health services and public sector or university-based researchers. Maximising the benefits from genomic medicine is also likely to require the involvement of commercial actors and governments as users, providers and/or regulators of data. Previous research suggests that the involvement of companies as users of genomic and health data may present concerns for data donors (Caulfield et al. 2014; Ipsos MORI 2016). However, there is little evidence on the prevalence of such concerns across national contexts, how members of the public discriminate between actors and how this relates to people’s willingness to donate their genomic and health data. Further, existing research suggests that ‘the public’ who are donating data and samples for genomic research are by no means a homogeneous group, and that responsible engagement should reflect the diverse groups which make up the donating public (Hoeyer 2010; Gaskell et al. 2013; Ipsos MORI 2016).

In this paper, we examine trust in data sharing among the general public, drawing from a representative sample of 8967 English-speaking members of the public from the UK, the USA, Canada and Australia. We explore levels of trust in the individual and organisational actors involved in the collection and sharing of genomic and health data. We identify subgroups within this large sample and examine the relationship between patterns of trust associated with these groups, demographic characteristics, including age, sex, educational level and religiosity, and experiences and expectations related to data. These include the willingness to donate data, experience of data breaches, concerns related to commercial and governmental use, and the perceived value of legal or regulatory action related to data governance.

## Methods

The findings presented are part of the ‘Your DNA, Your Say’ global online survey of public perceptions and values towards donating DNA and health data. Detailed methodological rationale for the study, design (and limitations), recruitment strategy and process of data collection have been published separately (Middleton 2018; Middleton et al. 2018b).

### Sample

Respondents were randomly recruited by email invite through the market research company Dynata, which adheres to the ESOMAR market research code of conduct. We collected completed surveys in the USA, Canada, United Kingdom (UK) and Australia (*n* = 8967). Participants were paid a small financial reward (< £1) for participating and due to the nature of recruitment there are no details on non-response rate. Respondents were recruited from Dynata’s nationally representative research panel in each country. However, the variables we collected within the study were oriented towards enabling comparison between countries. There is variability in how ethnicity and education data are collected between countries, while national levels of income also vary. Consequently, age and gender were retained as the two primary criteria on which we could match the YDYS population to census data (see Supplementary Material). Where sections of the population were under-represented, Dynata contacted additional respondents to more closely approximate census data.

### Measures

The cross-sectional, exploratory online survey can be accessed at www.YourDNAYourSay.org. It contains 29 questions and piloting showed it took approximately 15–20 min to complete.

#### Familiarity with DNA, genetics and genomics

Familiarity was ascertained based on two questions: “Are you familiar with DNA, genetics or genomics?” and “I’m familiar through my work, personal interests or family/medical history”. Participants were categorised as having “Personal” experience of genetics if they were familiar with DNA/genetics/genomics due to either having a genetic condition in their family, or through their work (e.g. genetic health professional or genetic researcher).

#### Trust in individuals and organisations

Participants were asked to identify what factors would influence their decision to donate their DNA and medical information. They were asked to indicate if they would trust the people below (multiple selections possible):

- My medical doctor.
- Any medical doctor in my country.
- Any researcher at a university in my country.
- Any researcher in a company in my country.
- The government of my country.

Response options were “I would generally trust”, “I’m just not sure” and “I would not generally trust”. These categories were used for descriptive analyses, with the latter two combined for the multivariable analyses. This distinguishes between those who have made a positive judgement about the trustworthiness of the entities concerned and others, reflecting our theoretical understanding of trust as an active ‘leap of faith’.

#### Donating DNA and medical information

Throughout the survey, participants were asked whether they would donate their “anonymous’’^1^ DNA and medical information for use by others. We asked participants to distinguish who they would allow to use their data, (a) medical doctors; (b) non-profit researchers; (c) for-profit researchers. Participants were classified as *willing to donate* if they answered “yes” to at least one of these questions, *unwilling to donate* if they answered “no” to all three, and *unsure* if they answered “unsure” to all three. This classification aimed to identify people who would be willing to donate in at least some circumstance.

#### Negative experiences online

To determine whether participants had experienced negative consequences relating to online access to personal information we asked: “Websites have variable levels of security. Have you had any negative experiences from your personal information being accessed online?”. Possible responses were “Yes”, “No”, “I’m not sure”. The latter two categories were collapsed for analysis purposes.

#### Concerns about specific harms

Participants were presented with a list of hypothetical harms that could occur as a result of their DNA information being accessed by others, and asked to indicate how concerned they felt about each of these (“Not bothered”, “Concerned”, “Very concerned”, “I’m not sure”). The list of hypothetical harms was based on pilot work, the academic literature and the experience of the authors who designed the survey. The full list has been described previously; for this research we focused on the following concerns that were considered relevant to trust in different organisations involved in using DNA and health data (Middleton et al. 2018a):

- My government potentially knowing something about me that I hadn’t chosen to tell them.
- Police potentially knowing something about me that I hadn’t chosen to tell them.
- Marketing companies targeting me to sell me products.
- Health or life insurance companies using the information to discriminate against me.

For analyses of these measures, the four categories of concern were collapsed into two; “Concerned” was combined with “Very concerned”, and “Not bothered” with “I’m not sure” to capture the existence of definite concerns,.

#### Influence of regulation on views of donation

Participants were asked “Would you be more comfortable donating your DNA and/or medical information if you knew there was a law in place to protect against being exploited?” with options “Yes”, “No”, “I’m not sure”. The latter two categories were collapsed for analysis.

#### Socio-demographics

Age was collected in 10-year categories from age 16 years onwards, but due to the lower number of responses in younger and older age categories, these were collapsed into three categories of “30 years and under”, “31–50 years” and “51 years and older” for analysis. Whether participants had children was determined by a “Yes” or “No” answer without specifying whether the children were biological or not. Relationship status was collected as “Divorced”, “Separated”, “Single”, “Widowed”, “Married/civil partnership/living together”, but all categories apart from the latter were collapsed for analyses.

We piloted how to collect ethnicity data, starting with the categories provided by the World Health Organization and the UK Office for National Statistics and adapting these based on feedback from pilot participants involved in survey development. The resultant ethnicity question in the final survey thus asked participants to self-identify as: white; Afro-European/African American, black; Hispanic; South Asian, Indian, Pakistani; East Asian Chinese, Japanese; Arabic, Central Asian; Other. Participants could also choose not to answer this question at all. In the analysis, due to low number of participants who self-identified as a member of a group other than “White” (less than 10% of the sample for each country), these were collapsed into a single “Non-White” category for analysis.

Highest level of education was categorised as “Tertiary”, “Secondary”, “Primary” or “Other” based on structured responses and also free-text descriptions of educational qualifications. This was collapsed to a binary indicator of tertiary education (vs no tertiary education) for multivariable analyses. Religiosity was determined by participants’ responses to the question “Independent of whether you attend religious services or not, would you say you are…?” with options “A religious person” or “Not a religious person”.

### Statistical analysis

Latent class analysis (LCA) identifies subgroups, or latent classes, within a sample using the pattern of respondents’ scores on the five trust variables. The aim is not to represent all possible combinations of characteristics, but to identify the main patterns present, assuming some measurement error (Lanza and Rhoades 2013; Lanza et al. 2013). In this case, the use of LCA aims to identify the number of groups that demonstrate distinct patterns of scores across the trust variables. To find the likely number of subgroups, models postulating increasing numbers of latent classes were sequentially fitted. Identification of each model was evaluated by refitting it using 100 sets of random starting values and inspecting the percentage that converged to the same solution (Lanza and Rhoades 2013; Lanza et al. 2013). The best-fitting model was selected by examining the Akaike information criterion (AIC), Bayesian information criterion (BIC) and entropy for each model and considering the size, distinctness and ease of interpretation of the classes identified (Nylund et al. 2007). This was informed by the class membership probabilities, the estimated proportion of the sample belonging to each class and the item-response probabilities for each class, which represent the likely values for the set of characteristics (i.e. probability of trusting each individual/organisation), given membership of a particular class.

Multinomial logistic regression models estimated via the ‘one-step’ approach were used to explore associations between individual covariates and subgroup membership (Feingold et al. 2013). Coefficient estimates for each class were combined with the known distribution of each covariate to estimate the probability of each covariate value conditional on latent class membership.

Models including adjustment for country of residence were fitted to examine the adjusted associations between class membership and (1) donating DNA and medical information; (2) knowledge of genetics; (3) negative experiences online; (4) concerns regarding use of DNA and medical information; (5) view on regulation and donation. All tests were two-tailed but given the number of models fitted, *α* = 0.05 was not appropriate. A Bonferroni corrected threshold would be *P* ≤ 0.007, although we present and interpret *P* values as measures of the strength of evidence for an association rather than simply applying a threshold for statistical significance (Greenland et al. 2016). Analyses were conducted in R (version 3.5.2) using the poLCA package (Linzer and Lewis 2011; R Core Team 2016).

## Results

### Sample characteristics

The total sample consisted of 8967 participants from the UK, USA, Canada and Australia. The socio-demographic characteristics of the sample are described in detail elsewhere, but are provided in Supplementary Table S1 for completeness (Middleton et al. 2018a).

Overall, participants were most likely to trust their medical doctor (see Table 1). Participants were less likely to trust the other individuals/organisations named, with researchers at a company being the individuals least likely to be trusted. There were limited missing data in the data set; 97% of the sample (*n* = 8659) were included in the complete case data set for the LCA.

**Table 1:** Number and percentage of participants expressing trust in doctors, researchers and government

Variable	Categories	Total no.	Total perc.
My medical doctor	No/unsure	2232	24.9
	Yes	6727	75
	Missing	8	0.1
Any medical doctor in my country	No/unsure	5393	60.1
	Yes	3562	39.7
	Missing	12	0.1
Any researcher at a university in my country	No/unsure	5899	65.8
	Yes	3060	34.1
	Missing	8	0.1
Any researcher at a company in my country	No/unsure	7768	86.6
	Yes	1192	13.3
	Missing	7	0.1
The government of my country	No/unsure	7251	80.9
	Yes	1709	19.1
	Missing	7	0.1

### Latent class analysis of trust in individuals and organisations

**Fig. 1:**
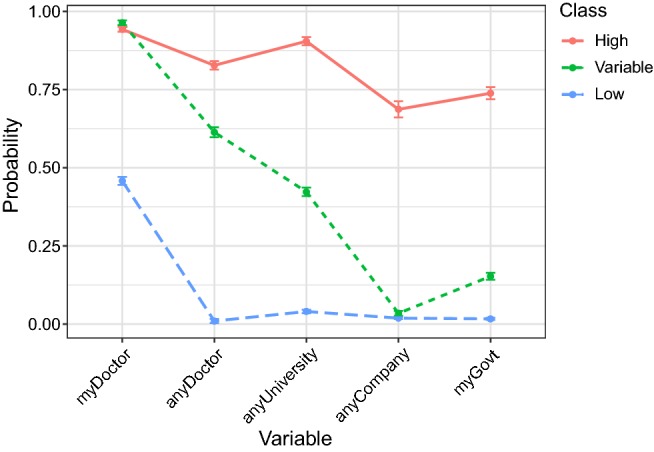
Item response probabilities for the 3-class model. Categories: myDoctor, my medical doctor; anyDoctor, any medical doctor in my country; anyUniversity, any researcher at a university in my country; anyCompany, any researcher at a company in my country; myGovt, the government of my country

Five latent class models (one to five classes) were fitted, although fit statistics did not unequivocally identify a best-fitting model (Supplementary Table S2). This discordance is not uncommon as the AIC and BIC have different strengths, despite the BIC generally selecting more parsimonious models and performing better for model selection in LCA of large samples (Nylund et al. 2007). The AIC was lowest for the five-class model, while the BIC was lowest for the four-class model, but both models potentially had identification problems (Supplementary Figure S1). The three-class model appeared to represent the best trade-off between AIC, BIC and entropy fit indices. Examination of the item-response probabilities confirmed that the subgroups identified in the three-class model had distinct characteristics (Fig. 1 and Supplementary Table S3), and that no class was too small (the smallest class contained 16% of the sample, approximately 1386 participants). These results indicated that the three-class model was most appropriate for these data.

The item-response probabilities (Fig. 1) suggest that the classes defined by the three-class model can be characterised as follows:

- *Low overall trust* (41% of the sample): Moderate trust in own medical doctor and no trust in any other individuals/organisations.
- *Variable trust* (43% of the sample): High levels of trust in medical professionals, moderate trust in university researchers and low trust in company researchers and own government.
- *High overall trust* (16% of the sample): High levels of trust in all individuals/organisations.

### Latent class characteristics

Participants in the *Variable Trust* class were more likely to be from the UK, while those in the *High Trust* class were more likely to be from the USA (see Table 2). Gender also appeared to differ across with classes, with the *High Trust* class members more likely to be male compared to the other two classes (0.63 compared to 0.5 for the *Variable Trust class* and 0.48 for the *Low Trust* class), and less likely to be over 50 (0.25 compared to 0.39 and 0.37). Those in the *High Trust* class were also more likely to have children (0.66 compared to 0.57 and 0.56) and to be more educated (they were less likely not to have a tertiary-level education, 0.33 compared to 0.42 and 0.47). Participants in the *High Trust* class were also substantially more likely to view themselves as a religious person (0.51 compared to 0.30 for the *Variable Trust* class and 0.39 for the *Low Trust* class). The *Low Trust* class were more likely to be divorced, single or widowed (0.42 compared to 0.36 of the *Variable Trust* class and 0.32 of the *High Trust* class). The *High Trust* class were the most likely to have personal experience of genetics (0.27 compared to 0.12 for the *Variable Trust* class and 0.09 for the *Low Trust* class). They were also slightly more likely to report being familiar with genetics (0.39 compared to 0.31 and 0.28 for the *Variable Trust* and *Low Trust* classes, respectively).

**Table 2:** Probability of characteristics related to demographics and knowledge of genetics given latent class membership

Variable	Category	Low trust	Variable trust	High trust
Country of residence	UK	0.33	0.48	0.26
	USA	0.25	0.14	0.35
	Canada	0.26	0.24	0.24
	Australia	0.16	0.14	0.15
Age	Over 50	0.39	0.37	0.25
	31–50	0.4	0.38	0.49
	30 and under	0.21	0.25	0.26
Gender	Male	0.5	0.48	0.63
Children	Yes	0.56	0.57	0.66
Tertiary eduction	No	0.47	0.42	0.33
Ethnicity	Non-white	0.18	0.1	0.16
Religiosity	A religious person	0.39	0.3	0.51
Relationship	Divorced/single/widowed	0.42	0.36	0.32
Genetics knowledge	Unfamiliar	0.63	0.57	0.34
	Familiar	0.28	0.31	0.39
	Personal	0.09	0.12	0.27

### Associations with latent class membership

Based on the multinomial logistic regression model, adjusted for country of residence, willingness to donate DNA and medical information was most strongly associated with membership of the *High Trust* class (OR 22.5, 95% CI 15.5–32.5; see Table 3), although there was also a strong association with the *Variable Trust* class (OR 6.2, 95% CI 5.2–7.4). Being unsure about donating was not associated with a particular class. The association between having had a negative experience online and trust profile varied substantially. Interestingly, a negative experience was associated with reduced odds of membership of the *Variable Trust* class (OR 0.67, 95% CI 0.56–0.81), but increased odds of belonging to the *High Trust* class (OR 3.1, 95% CI 2.6–3.6).

**Table 3:** Multinomial logistic regression result for associations of latent class membership with willingness to donate, and experiences and concerns related to data use

Variable	Category	Latent class			
		Variable trust		High trust	
Donation	Unwilling	Ref.		Ref.	
	Willing	6.19 (5.16–7.43)	< 0.0001	22.47 (15.53–32.51)	< 0.0001
	Unsure	1.51 (1.22–1.86)	0.02	1.64 (1.02–2.62)	0.12
Negative experience online	No/unsure	Ref.		Ref.	
	Yes	0.67 (0.56–0.81)	0.006	3.07 (2.63–3.59)	< 0.0001
Concern—government	No/unsure	Ref.		Ref.	
	Yes	1 (0.87–1.14)	0.95	0.51 (0.44–0.58)	< 0.0001
Concern—police	No/unsure	Ref.		Ref.	
	Yes	0.74 (0.65–0.84)	0.003	0.63 (0.55–0.72)	0.001
Concern—marketing	No/unsure	Ref.		Ref.	
	Yes	1.94 (1.63–2.3)	< 0.0001	0.72 (0.62–0.84)	0.006
Concern—insurance	No/unsure	Ref.		Ref.	
	Yes	1.79 (1.51–2.12)	0.001	0.74 (0.63–0.88)	0.012
Laws around donation	No/unsure	Ref.		ref.	
	Yes	6.61 (5.7–7.66)	< 0.0001	16.18 (13.25–19.77)	< 0.0001

Participants who expressed concern about the government knowing something about them if their DNA was linked to other personal information had substantially lower odds of belonging to the *High Trust* class (OR 0.51, 95% CI 0.44–0.58), but this concern did not distinguish between members of the *Variable Trust* and *Low Trust* classes. Participants who were concerned about police use of this information had much lower odds of belonging to the *Variable Trust* or *High Trust* classes (OR 0.75, 95% CI 0.65–0.84 and OR 0.63, 95% CI 0.55–0.72 respectively). Concern regarding the use of this information for marketing or insurance purposes was associated with much greater odds of belonging to the *Variable Trust* class (OR 1.96, 95% CI 1.63–2.3 and OR 1.79, 95% CI 1.51–2.12 respectively) and somewhat lower odds of belonging to the *High Trust* class (OR 0.72, 95% CI 0.62–0.84 and OR 0.74, 95% CI 0.63–0.88). Feeling reassured by laws preventing exploitation of donated information was associated with much greater odds of belonging to the *Variable Trust* and *High Trust* classes (OR 6.61, 95% CI 5.7–7.7 and OR 16.2, 95% CI 13.3–19.8 respectively).

## Discussion

Overall, the extent to which respondents trust different actors with their genomic and health data varies. Trust is strongest in individuals’ own doctors and lowest for other actors, particularly for companies and governments. These differences are consistent with other studies of public attitudes to commercial and government use of health and genomic data across the countries investigated here (Caulfield et al. 2014; Critchley et al. 2015; Ipsos MORI 2016; Garrison et al. 2016). They reaffirm the importance of trust in doctors and the gatekeeping role played by individual’s own doctors in supporting the development of large-scale data sharing initiatives (Kelly et al. 2015). It presents challenges and suggests the need to support dialogue about the role of research partnerships between public and private sectors.

The public are often seen as supportive of biomedical research and trusting of those who conduct it (Lipworth et al. 2009). However, concerns about public trust reflect a perception that the public are potentially distrustful of many of the actors involved in data sharing. Our analysis suggests that as we build societal conversations about the sharing and use of genomic data, it is important to recognise that the general public is not a uniform group with congruent interests (Resnik 2011; Caulfield et al. 2014). We have identified three subgroups characterised by their level of trust across the actors presented. Further, we have described differences in how these groups approach questions of data donation and sharing.

We identified a large group of the public who do not trust any group other than their own doctor with their DNA or health data. This distrustful, or at least sceptical, population represents a challenge for efforts to establish the trustworthiness of entities involved in genomic data sharing. Typically, membership of this subgroup is associated with lower levels of willingness to donate data and increased odds of concern about the use or misuse of DNA data by police and governments. Membership of this group overlaps in terms of age, education and having children with those who are unwilling to donate their data (Middleton et al. 2018a). The low numbers of respondents from non-white ethnic groups limits the ability to identify differences related to ethnicity between *Low Trust* and the *High* and *Variable Trust* groups. There may be value of further exploration of the relationship between trust and donation across population subgroups.

Membership of the *Variable Trust* class was also associated with greater concern about government use of data, reflecting a shared lack of trust in government across these subgroups. Moreover, members of the *Variable Trust* group were also more concerned about the use of DNA or health data for insurance and marketing purposes, reflecting their lower level of trust in companies. It seems that, while legal reforms to address data sharing and the exploitation of donated information had little value for the low trust group, such laws appear to be more reassuring for the variable trust group. This suggests the potential for regulation to increase trust in data sharing among a sizeable subgroup of the population, but also that the efficacy of such approaches depends on prior confidence in the ability of actors to manage risks to data donors. Such findings align with those studies that find that trust in science is greater among those with trust in other national institutions (Wellcome Trust 2019). This group, then, might be characterised as adopting a position of critical trust, in which a reliance on institutions to govern data sharing and use data responsibly is combined with a critical evaluation of their ability to do so (Walls et al. 2004).

Echoing previous findings, we find that membership of the *High Trust* subgroup is strongly associated with greater willingness to donate DNA and health data (Tomlinson et al. 2015). This group is more likely to be under 50 years, male, have children, hold religious beliefs, be from the USA and have a tertiary education and less likely to be single, divorced or widowed. They are also more likely to have personal familiarity with genetics, echoing previous findings that those who interact most frequently with health services are those most likely to support data sharing, including with the private sector (Ipsos MORI 2016). Previous work on genetics has also suggested that men may be more accepting of genetic technologies and to perceive fewer risks (Connor and Siegrist 2010). This subgroup is more likely to have trust in individuals and organisations and also to have wider confidence in systems to manage and mitigate risks associated with sharing genomic data, as suggested by the much greater odds of this group supporting the value of laws to protect their data (Luhmann 2000). The characteristics of this group might also be indicative of an association between greater levels of social capital, in terms of the perceived agency of individuals in society and their connectedness to social organisations, and trust. The finding that this population comprises also those who are most likely to have had negative experiences related to access of their personal information data online may point to the resilience of systemic confidence in this group—and a greater willingness to take the risky ‘leap of faith’ associated with trusting in data sharing arrangements.

## Limitations

Exploratory cross-sectional online surveys have limitations in that they capture an attitude or perception about intended behaviour at a single time point. While attitudes are a useful guide to behaviour, they may not directly translate. Hence, there is some evidence that people are more restrictive in hypothetical data sharing choices than those they adopt in practice (Oliver et al. 2012).

Overall limitations of the study and design have been published separately (Middleton et al. 2018c). Our results do not represent all English-speaking people around the world, nor should our findings be extrapolated to indicate views of all people from the USA, Canada, the UK and Australia. The survey was not designed to test a particular approach to supporting the development of public trust, but to generate hypotheses related to the perceptions and characteristics of the public as they relate to genomic data sharing. The cross-sectional data presented here suggest potentially interesting relationships between previous experiences of data-related problems and trust, and in the ability of regulation to modify trust in genomic data sharing. Further work might explore whether this is affected by the provision of detail on how legal protections work. Future research might also investigate the relationship between data controversies and trust over time, and how it is affected by shifts in regulatory landscapes, including the EU General Data Protection Regulation.

## Conclusions

This analysis adds to our understanding of how and to whom public trust is given in genomic data sharing. It reinforces the importance of acknowledging and being transparent about different users of genomic and health data, and of building on individual interpersonal relationships of trust. By identifying different groups within the study population, the analysis contributes to understanding how trust varies, how this relates to specific areas of concern associated with the use of genomic data by different actors and the potential of legal reform to mitigate these concerns.

The study findings are particularly pertinent as genomic data sharing moves from a research-focussed enterprise to a wider societal endeavour. Whereas those members of the public involved in research may be those most trusting of researchers and clinicians, larger-scale efforts will inevitably draw those who are more sceptical into contact with genomic data sharing. In such circumstances, it is valuable to recognise the diversity of trust, the limits of policy and legislative action in reinforcing trust; and to consider how to engage with ambivalence in building approaches to data collection, sharing and use (Cunningham-Burley 2006). Finally, the study points to the importance of considering the range of social and cultural contexts within which data sharing occurs, as genomics enters the global clinical mainstream.

## Electronic supplementary material

Below is the link to the electronic supplementary material.

Supplementary material 1 (PDF 118 kb)

## Footnotes

1 Here we mean ‘de-identified’, but pilot work showed that ‘anonymous’ was more easily understood by participants. A glossary definition within the survey explained ‘anonymous’ as: ‘removal of personal information such as name and date of birth. It is questionable as to whether DNA information can ever be truly anonymous as our DNA code is unique to us and thus, in itself, could be used to identify us. However, in the circumstances we are exploring here, by making DNA and medical information 'anonymous', we mean detaching personal identifiers from it.’.
