# Supplementary material for: Trust in genomic data sharing among members of the general public in the UK, USA, Canada and Australia
Source: Hum Genet. 2019 Sep 17;138(11):1237–46. doi: 10.1007/s00439-019-02062-0 (PMC6874520; doi:10.1007/s00439-019-02062-0)
Supplement: Supplementary file 1 — Supplementary material 1 (PDF 118 kb) [file 439_2019_2062_MOESM1_ESM.pdf]

Trust in genomic data sharing among members of the general public in the UK, USA, Canada and Australia

Supplementary Appendix

Milne *et al.*

Contents

1 Comparison with national census data **2**

2 Descriptive statistics for full YDYS English-speaking sample **3**

3 Latent Class Analysis Model Fitting **4**

1 Comparison with national census data

The YDYS sample was compared with the most recent available census data from each country:

- UK (2011 data; www.ons.gov.uk/census/2011census [accessed 13/09/2017])
- USA (2015 data; www.census.gov [accessed 13/09/2017])
- Canada (2016; http://www12.statcan.gc.ca/census-recensement/index-eng.cfm [accessed 13/09/2017])
- Australia (2016; http://www.abs.gov.au/websitedbs/censushome.nsf/home/2016 [accessed 13/09/2017])

Table S1: Comparison of YDYS samples with most recent census data from each country: proportion of sample in age and gender categories by country of residence.

**Variable**	**Categories**	**UK**		**USA**		**Canada**		**Australia**	
		YDYS	Census	YDYS	Census	YDYS	Census	YDYS	Census
Age	30 and under	27	24.8	17.6	25.5	25.8	22.4	21.1	18
	31-40	20.9	16.4	30.3	16.2	22.3	15.8	20.4	19
	41-50	17.9	17.9	15.8	15.8	18.3	15.8	18	18
	51-60	17	14.6	20	16.8	16.5	18.1	16.4	17
	Over 60	17.2	26.3	16.2	25.7	16.9	28.1	24.2	28.2
Gender	Female	50.3	50.8	51.3	50.7	41.5	51.3	51.8	51
	Male	49	49.2	48.1	49.3	57.1	48.7	47.6	49

2 Descriptive statistics for full YDYS English-speaking sample

Table S2: Descriptive statistics for full sample

Variable	Categories	Number	Percentage
Country	United Kingdom	3316	37
	United States	1992	22.2
	Canada	2257	25.2
	Australia	1402	15.6
Age	Over 50	3252	36.3
	31-50	3616	40.3
	30 and under	2091	23.3
	Missing	8	0.1
Gender	Female	4328	48.3
	Male	4574	51
	Missing	65	0.7
Education	Yes	5173	57.7
	No	3784	42.2
	Missing	10	0.1
Ethnicity	White	7539	84.1
	Non-White	1315	14.7
	Missing	113	1.3
Religiosity	Not a religious person	5609	62.6
	A religious person	3349	37.3
	Missing	9	0.1

3 Latent Class Analysis Model Fitting

Table S3: Model fit indices for Latent Class Analysis models with one to five classes.

Number of classes	Log-likelihood	BIC	AIC	Entropy
1	-23989.89	48025.13	47989.78	NA
2	-21096.77	42293.32	42215.54	0.68
3	-20544.56	41243.31	41123.12	0.65
4	-20468.04	41144.70	40982.08	0.61
5	-20458.52	41180.09	40975.05	0.70

Figure S1: Model identification plot for Latent Class Analysis models with two to five classes

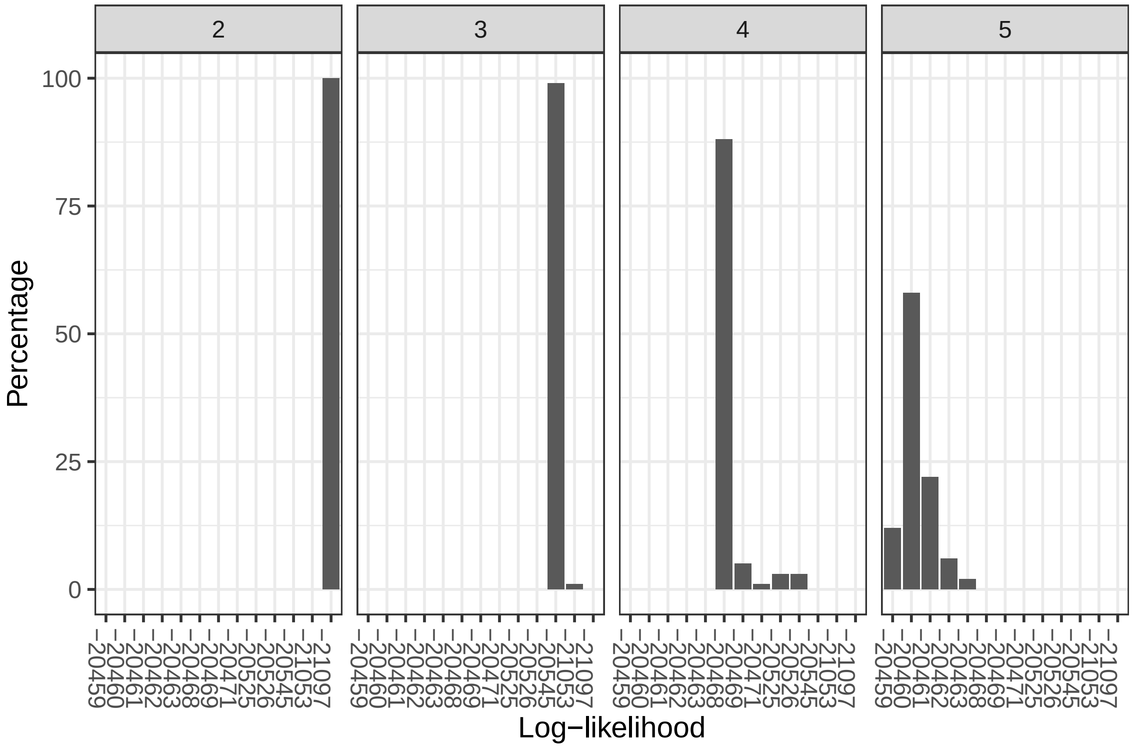

Table S4: Class membership and item response probabilities for 3-class model.

Category	Class 1	Class 2	Class 3
Class membership sample proportion	0.41	0.43	0.16
My medical doctor	0.46	0.96	0.95
Any medical doctor in my country	0.01	0.61	0.83
Any researcher at a university in my country	0.04	0.42	0.91
Any researcher at a company in my country	0.02	0.04	0.69
The government of my country	0.02	0.15	0.74

Table S5: Class membership and item response probabilities for 4-class model.

Category	Class 1	Class 2	Class 3	Class 4
Class membership sample proportion	0.34	0.05	0.48	0.14
My medical doctor	1.00	0.74	0.46	0.99
Any medical doctor in my country	0.65	0.41	0.01	0.91
Any researcher at a university in my country	0.43	0.45	0.03	0.97
Any researcher at a company in my country	0	0.34	0.01	0.70
The government of my country	0.13	0.38	0.01	0.76
